# Fundal Placenta Accreta: A Rare Presentation and Comprehensive Review

**DOI:** 10.7759/cureus.78726

**Published:** 2025-02-08

**Authors:** Adil Elghanmi, Fadila Kouhen, Leila Abdallaoui Maane, Karima Fichtali, Bouchra Ghazi

**Affiliations:** 1 Gynecology and Obstetrics, Immunopathology-Immunotherapy-Immunomonitoring Laboratory, Faculty of Medicine, Mohammed VI University of Health and Sciences (UM6SS), Casablanca, MAR; 2 Gynecology and Obstetrics, Mohammed VI International University Hospital, Bouskoura, MAR; 3 Laboratory of Neuro-Oncology, Oncogenetics, and Personalized Medicine, Mohammed VI University of Health Sciences (UM6SS), Casablanca, MAR; 4 Radiotherapy, Cheikh Khalifa International University Hospital, Casablanca, MAR; 5 Obstetrics and Gynecology, Cheikh Khalifa International University Hospital, Mohammed VI University of Health Sciences (UM6SS), Casablanca , MAR; 6 Obstetrics and Gynecology, Immunopathology-Immunotherapy-Immunomonitoring Laboratory, Faculty of Medicine, Mohammed VI University of Health Sciences (UM6SS), Casablanca, MAR; 7 Obstetrics and Gynecology, Mohammed VI International University Hospital, Bouskoura, MAR; 8 Immunology, Immunopathology-Immunotherapy-Immunomonitoring Laboratory, Faculty of Medicine, Mohammed VI University of Health Sciences (UM6SS), Casablanca, MAR; 9 Reproductive Medicine, Mohammed VI International University Hospital, Bouskoura, MAR

**Keywords:** cesarean section risk factors, fundal placenta accreta, mri in placental disorders, obstetric hemorrhage, placenta accreta spectrum (pas)

## Abstract

Fundal placenta accreta is a rare and challenging condition characterized by abnormal placental adherence to the myometrium, typically in the upper uterine segment. This case report describes a 35-year-old woman with a history of two previous cesarean sections, who presented at 19 weeks gestation with antepartum hemorrhage and was diagnosed with fundal placenta accreta. Diagnostic imaging, including ultrasound and MRI, revealed a centroplacental hematoma and signs of myometrial invasion, which were confirmed histopathologically after emergency extraction and postpartum management. Placenta accreta presents significant risks, primarily hemorrhagic, and requires careful diagnosis and management. Early detection using advanced imaging techniques, such as Doppler ultrasound and MRI, is crucial for planning treatment. Conservative and radical surgical options, including cesarean hysterectomy, must be considered depending on the severity of the condition.

## Introduction

Fundal placenta accreta represents a rare and challenging clinical entity within the placenta accreta spectrum (PAS), a group of disorders characterized by abnormal adherence of the placenta to the uterine myometrium [1]. PAS arises due to a defective decidualization of the basal decidua, resulting in either partial or complete absence of the decidual interface that normally separates the chorionic villi from the myometrium [2]. This pathological adherence predisposes to abnormal placental implantation and invasive placental growth.

The majority of reported placenta accreta cases are associated with a history of uterine surgery, particularly cesarean deliveries, which create a vulnerable, scarred myometrium prone to abnormal implantation [3]. In contrast, fundal placenta accreta, a variant occurring in the upper uterine segment away from previous uterine scars, remains poorly understood due to its rarity and atypical presentation [4].

Histopathological studies have revealed that placenta accreta is frequently accompanied by chronic basal inflammation and an increased lymphocytic infiltrate, which may further exacerbate the pathological attachment of the placenta [5]. Advances in imaging, particularly ultrasound and magnetic resonance imaging (MRI), have significantly enhanced the diagnosis of PAS. However, the diagnosis of fundal placenta accreta presents unique challenges due to its unusual location and lack of a scar-related risk factor, often leading to delayed recognition and increased peripartum complications [6].

This case report highlights a rare presentation of fundal placenta accreta, illustrating its clinical features and radiological findings, while also reviewing the current literature to provide insights into its diagnosis, pathophysiology, and management.

## Case presentation

A 35-year-old woman, gravida 3, para 2, at 19 weeks of gestation was referred for evaluation of antepartum hemorrhage. Her obstetric history included two prior cesarean sections, each involving a uterine incision. On initial assessment, she was in good general health with stable vital signs, including normal blood pressure and no signs of fever. Clinical examination revealed slight pallor, although she reported no uterine contractions.

**Figure 1:**
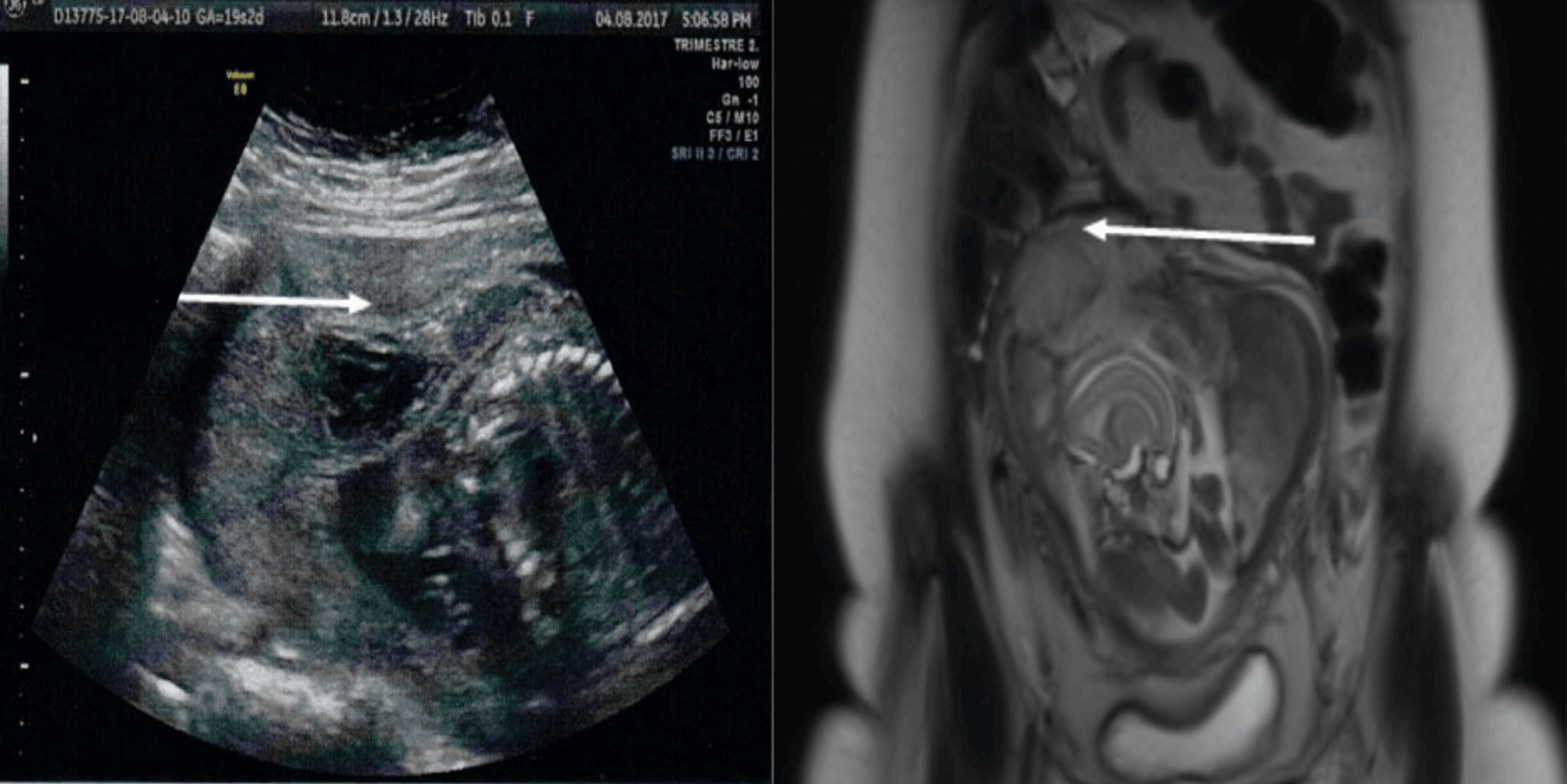
(Left) Central placental hematoma at 19 weeks of gestation and appearance of placenta accreta; (right) MRI sagittal view showing signs of fundal placenta accreta. MRI, magnetic resonance imaging

A speculum examination revealed active bleeding originating from the endocervix. The cervix was firm, long, and posteriorly positioned. Obstetrical ultrasonography confirmed a progressing singleton pregnancy at 19 weeks and five days of gestation (Figure 1).

Additional findings included oligohydramnios, which caused elevated umbilical artery resistances, and the presence of a centroplacental hematoma. These findings raised a strong suspicion of fundal placenta accreta, an uncommon form of placenta accreta localized in the uterine fundus.

MRI was performed to confirm the diagnosis (Figure 1). The imaging showed an anterior centroplacental hematoma measuring 6 cm in its longest dimension. The hematoma appeared hyperintense on T1- and T2-weighted images, with no retroplacental component identified. The placenta displayed heterogeneity, with prominent hyperintense intraplacental vascular lakes, further supporting the diagnosis of placenta accreta. Additional signs of myometrial invasion, including thinning and irregularity of the myometrial interface, were also noted, reinforcing the suspicion of fundal placenta accreta.

During follow-up, the patient experienced persistent vaginal bleeding, accompanied by progressive signs of deglobulization, as evidenced by a significant decline in hemoglobin levels. Her hemoglobin concentration dropped from an initial 10 to 8 g/dL, and subsequently to 6 g/dL, indicative of substantial blood loss.

Given the clinical deterioration, an emergency extraction was performed. The status of the fetus was carefully evaluated, and due to the severe maternal complications, the fetus was determined to be non-viable at the time of the procedure. Informed consent was obtained for the second-trimester abortion and hysterotomy, with clear communication regarding the risks involved, including excessive hemorrhage and the potential need for a hysterectomy. Upon uterine incision, hemorrhagic amniotic fluid was noted. A slow intravenous injection of 5 IU oxytocin was administered, and gentle traction was applied to the umbilical cord, facilitating the manual removal of placenta. A uterine-sparing hysterectomy was performed, and the uterine incision was sutured with hemostatic sutures in the placental bed; however, extensive bleeding from the placental bed ensued. Hemostatic measures, including a continuous infusion of 10 IU oxytocin and the administration of sulprostone, a prostaglandin analog, successfully controlled the hemorrhage.

Postoperatively, the patient was closely monitored in the recovery room, where no further bleeding was observed. She was subsequently transferred to the intensive care unit for 24 hours of observation, which remained uneventful. The patient was discharged on postoperative day 5 in stable condition.

At a follow-up visit on postoperative day 12, the patient showed no signs of complications, and her symptoms had resolved completely. Histopathological examination of the placenta confirmed the diagnosis of placenta accreta, characterized by abnormal trophoblastic invasion of the myometrium without intervening decidua.

## Discussion

Fundal placenta accreta is a rare and underreported variant of the PAS, where abnormal placental adherence occurs in the upper segment of the uterus. Unlike the more commonly reported anterior and lower uterine segment accreta associated with prior cesarean sections or uterine surgeries, fundal placenta accreta is often asymptomatic, making its diagnosis particularly challenging [7]. This condition is typically discovered incidentally during cesarean deliveries or in the event of significant obstetric complications, such as postpartum hemorrhage.

The pathophysiology of fundal placenta accreta remains poorly understood but shares common mechanisms with other PAS disorders. These include defects in the decidua basalis, excessive trophoblastic invasion, and impaired immune regulation, particularly a reduction in decidual natural killer (dNK) cells [8]. While most cases of placenta accreta are linked to prior uterine trauma, the absence of fundal lesions in some cases, as observed in this report, suggests that other contributing factors, such as immune dysregulation, might play a critical role.

Diagnosis of fundal placenta accreta relies heavily on imaging modalities, given its atypical presentation. Gray-scale ultrasound is often the first-line diagnostic tool, but its sensitivity is reduced for fundal locations due to the challenges of visualizing the upper uterine segment. Key findings on ultrasound include intraplacental lacunae, thinning of the myometrium, and irregularity of the uterine contour. Doppler ultrasound can further aid in the diagnosis by identifying abnormal vascularization extending into the myometrium [9]. However, MRI is particularly valuable for fundal placenta accreta due to its ability to provide detailed three-dimensional imaging of the uterine anatomy. MRI findings include heterogeneous placental signal intensity, irregular thinning of the myometrium, and extension of abnormal vasculature into adjacent tissues [10].

Management of fundal placenta accreta presents unique challenges due to its atypical location. Radical approaches, such as cesarean hysterectomy, are often the standard of care, particularly in cases with extensive invasion or life-threatening hemorrhage [11]. However, conservative management, including uterine-sparing procedures, has been employed successfully in selected cases. These approaches may involve leaving the placenta in situ, arterial embolization, or placental bed suturing. While conservative management can preserve fertility, it carries risks such as delayed postpartum hemorrhage, infection, and thromboembolism, necessitating close monitoring and multidisciplinary care [12].

## Conclusions

This case highlights the importance of multidisciplinary collaboration and prompt intervention in managing fundal placenta accreta. Conservative approaches, while potentially preserving fertility, require meticulous monitoring to mitigate the risks of infection, thromboembolism, and delayed hemorrhage. Radical surgical interventions, such as cesarean hysterectomy, remain the gold standard in cases of severe invasion or uncontrollable bleeding. Further research into the pathophysiology, risk factors, and long-term outcomes of fundal placenta accreta is warranted to guide evidence-based management and improve maternal outcomes.
